# Long-term real-world effectiveness of carbidopa/levodopa enteral suspension after 36 months of treatment initiation in advanced Parkinson's disease in the US: final results from the PROviDE study

**DOI:** 10.1016/j.prdoa.2026.100488

**Published:** 2026-07-26

**Authors:** Rajesh Pahwa, Stuart H. Isaacson, David Cella, Sandeep Thakkar, Prasanna L. Kandukuri, Connie H. Yan, Pavnit Kukreja, Irene Pan, Jae Lee, Jason Aldred

**Affiliations:** a University of Kansas Medical Center, Kansas City, KS, USA; b Parkinson's Disease and Movement Disorders Center, Boca Raton, FL, USA; c Northwestern University, Chicago, IL, USA; d OC Parkinson's Clinic, Irvine, CA, USA; e AbbVie Inc., North Chicago, IL, USA; f United BioSource LLC, King of Prussia, PA, USA; g Selkirk Neurology, Spokane, WA, USA

**Keywords:** Advanced Parkinson's disease, Carbidopa/levodopa enteral suspension, Deep brain stimulation, Patient-reported outcomes, Quality of life

## Abstract

**Introduction:** Clinical trials and observational studies have demonstrated that carbidopa/levodopa enteral suspension (CLES) significantly improves advanced Parkinson's disease (aPD) symptoms. This study examined the real-world, long-term impact of CLES on patient-reported outcomes (PROs) in people with aPD.

**Methods:** Pursuing Real-World Outcomes via Duopa Ecosystem (PROviDE) was a 36-month prospective, observational study evaluating the effectiveness of CLES in patients with aPD in the US. PROs were collected at baseline, 3 months, and every 6 months after CLES initiation and included OFF-time, Treatment Satisfaction Questionnaire for Medication-9 (TSQM-9), and Parkinson's Disease Questionnaire-8 (PDQ-8) assessing PD-related quality of life (QoL). Longitudinal treatment effects were evaluated using mixed-effect models.

**Results:** Of 104 patients, 47 (45.2%) provided 36 months of data and were evaluated in the final mixed-model analysis. Patients mean standard deviation (SD) age was 67.0 (8.7) years with a mean (SD) PD diagnosis of 11.0 (6.2) years. Compared with baseline, CLES resulted in statistically significant and clinically meaningful improvements in hours of OFF-time as early as month 3 (mean: −2.7 h/day, *p* < .0001) and sustained at month 36 (−1.7 h/day, *p* = .004). Improvements were also observed in treatment effectiveness (≥19 points, all p < .0001), global satisfaction with treatment and convenience of therapy (≥10 points each; all *p* < .001) over 36 months. A statistically significant improvement in PDQ-8 was observed as early as month 3 (−4.1, *p* = .001) with no worsening over time.

**Conclusion:** Long-term treatment with CLES provides significant and clinically meaningful reduction in OFF-time, patient's treatment satisfaction, and maintained QoL in the real-world setting.

## Highlights

• Long-term, real-world US study evaluating effectiveness of CLES in aPD.
• CLES provided sustained and clinically meaningful reductions in OFF-time.
• Treatment satisfaction and PD-related QoL improved with CLES.
• CLES led to significant improvements at 3 months and sustained through 36 months.
• CLES may be a viable treatment option for patients previously treated with DBS.

## 1 Introduction

### 1.1 Background

Parkinson's disease (PD) is a neurodegenerative disorder with progressive motor and nonmotor symptoms, which increasingly and negatively impact health-related quality of life (HRQoL) [1]. In the early stages of PD, oral levodopa remains the gold standard therapy and typically provides robust clinical benefit for symptoms. However, as PD advances, the narrowing therapeutic window, delayed gastric emptying, and short half-life of oral levodopa result in decline of efficacy and reliability of oral levodopa leading to development of motor fluctuations that become challenging to adequately manage [2]. To address these challenges, treatment regimens typically include higher and/or more frequent dosing, or addition of adjunctive agents; however, these approaches can result in increased pill burden, reduced adherence, and may still leave symptoms inadequately controlled. When optimized oral and adjunctive therapies become insufficient, patients with advanced PD (aPD) may transition to device-aided therapies (DATs). Deep brain stimulation (DBS) and continuous infusion treatments, such as carbidopa/levodopa enteral suspension (CLES), known as levodopa-carbidopa intestinal gel (LCIG) outside the US, or the 24-h continuous subcutaneous infusion of foslevodopa/foscarbidopa (LDp/CDp) or apomorphine achieve more consistent symptom control and improved HRQoL [3].

CLES was approved in the US in 2015 for the treatment of motor fluctuations in people with aPD [4], [6], [7]. CLES is delivered into the jejunum via percutaneous endoscopic gastrojejunostomy and a portable external pump, administered continuously over a 16-h waking day and provides stable plasma levodopa levels through a gel combination of carbidopa/levodopa [8]. Clinical trials [7], [9] and observational studies [2], [10], [11] have demonstrated that CLES decreases OFF-time, and improves QoL [12]. Additionally, the use of CLES in patients previously treated with DBS has been shown to be effective in reducing OFF-time while improving QoL; however, this is limited to small case studies and retrospective studies [8], [13], [14], [15].

While the efficacy of CLES is well documented [6], US-specific real-world data on long-term effectiveness of CLES therapy are sparse. Pursuing Real-world Outcomes via Duopa Ecosystem (PROviDE) is a prospective, observational study that evaluated the effectiveness of CLES in patients with aPD in the US, including a subgroup of patients with previous self-reported DBS therapy. The objective of this study was to examine the real-world, long-term effects of CLES on patient-reported outcomes (PROs), including OFF-time, treatment satisfaction, and PD-related QoL over 36 months.

## 2 Methods

### 2.1 Study design and patient population

PROviDE is a prospective, observational, longitudinal 36-month study assessing outcomes in patients with aPD in the US. Patients who were initiating CLES, and were enrolled in the DuoConnect Hub, a personalized support program available for patients, caregivers, and healthcare providers were eligible to participate. Patients had to be English speaking and ≥ 18 years of age with a diagnosis of aPD. Patients with current or previous enrollment in a clinical or observational CLES study, or in hospice care were excluded. Patients were prescreened for eligibility and recruited between January 15, 2017, and June 30, 2018. The study was reviewed and approved by a central institutional review board. All data collected in this study were de-identified and strictly confidential in accordance with local, state, and federal law.

### 2.2 Outcome measures

Baseline patient sociodemographic and clinical characteristics were collected by a study coordinating center representative. Outcome assessments, in the form of patient-reported questionnaires, were collected at baseline, 3 months after CLES initiation, and every 6 months thereafter up to 36 months. Follow-up assessments were completed based on participant preference either by telephone or online. For online assessments, participants entered responses directly into the secure web-based system; for telephone assessments, a study representative entered participant responses into the electronic system on their behalf.

The primary outcome was change in patient-reported OFF-time hours from baseline. Daily hours of OFF-time were measured by the Modified Movement Disorder Society-Unified Parkinson's Disease Rating Scale (MDS-UPDRS) Part IV item 39 and normalized to a 16-h waking day using PD diaries [17]. Additional PROs included treatment satisfaction, measured using the abbreviated Treatment Satisfaction Questionnaire for Medication (TSQM-9) across 3 domains: effectiveness, convenience, and global satisfaction. Each domain is scored on a scale of 0 to 100, with higher scores indicative of greater satisfaction [16]. PD-related QoL was measured using the Parkinson's Disease Questionnaire – Short Form (PDQ-8), a validated 8-item questionnaire measuring health across 8 domains; the PDQ-8 summary index ranges from 0 (normal) to 100 (worst disability) [17]. Additional questionnaires addressed freezing of gait, sleep, dyskinesia, and fatigue. The Freezing of Gait Questionnaire–self-administered (FOGQsa) version is a validated, 6-item questionnaire with total scores ranging from 0 to 24 (higher scores indicating more severe FOG) [18]. The Parkinson's Disease Sleep Scale (PDSS-2) was used to assess sleep symptoms, which is a self-administered questionnaire on PD-related sleep and nocturnal disturbances [19]. Parkinson's disease Dyskinesia Scale (PDYS-26) is a 26-item patient-based questionnaire designed to assess the impact of dyskinesia on activities of daily living (ADL) in PD [20]. The Patient-Reported Outcome Measurement Information System-Fatigue Short Form (PROMIS-F SF) (higher scores indicating higher fatigue) [21]. Similar outcomes were additionally assessed in a subgroup of participants with prior DBS.

### 2.3 Statistical analysis

The final analysis is based on patients who completed baseline and PRO assessments and had CLES supply for at least 80% of the follow-up period. The use of an 80% Proportion of Days Covered (PDC) criterion was evaluated for all visits until the last visit prior to discontinuation (defined as last shipment date in DuoConnect + days' supply). This 36-month final data was summarized by descriptive statistics, paired *t*-tests and repeated measure models, adjusted for age, gender, ability to walk, number of years since PD diagnosis, and treatment discontinuation. The last observation carried forward (LOCF) method was applied to account for missing data from visits between baseline and patient's last visit. Analyses were performed using SAS v9.4 or higher (SAS Institute Inc., Cary, NC, USA).

## 3 Results

### 3.1 Demographics and clinical characteristics

**Fig. 1:**
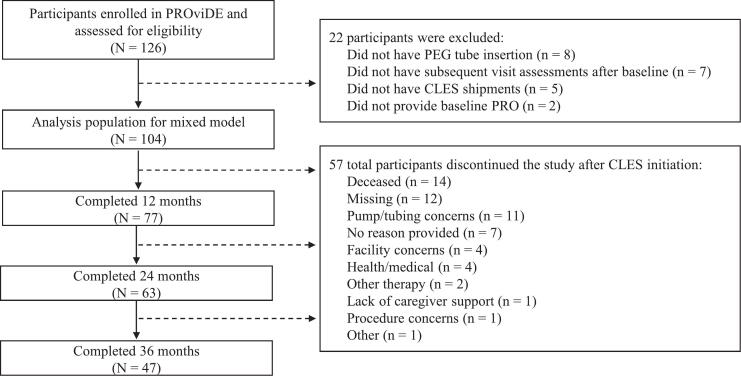
Patient disposition. Abbreviations: CLES, carbidopa/levodopa enteral suspension; PEG, percutaneous endoscopic gastrostomy; PRO, patient-reported outcome.

A total of 126 patients were enrolled in the PROviDE study and 104 met eligibility criteria and were included in the analytic sample at baseline (Fig. 1). Of those, 57 (54.8%) patients discontinued the study and 47 (45.2%) provided both baseline and 36-month data and made up the final analytic sample and 80% PDC on medication use. Table 1 presents baseline demographic and clinical characteristics of the total analytic sample. At baseline, the mean age was 67.0 years and predominantly male (*n* = 68, 65.4%). The mean years since PD diagnosis was 11.0 years. Most patients (*n* = 77, 74.0%) reported regularly using a walking aid, such as a cane or walker. Approximately one-fifth (*n* = 22, 21.2%) of patients reported previous use of DBS.

**Table 1:** Baseline demographic and clinical characteristics of the analysis population of the PROviDE study.^a^, ^c^

Characteristic	Patients *N* = 104
Age, years, mean (SD)	67.0 (8.7)
Male, n (%)	68.0 (65.4)
Years since PD diagnosis, mean (SD)	11.0 (6.2)
Hours of OFF-time,^b^ mean (SD)	5.7 (3.1)
Hours of dyskinesia per day, c mean (SD)	2.9 (2.8)
Treatment satisfaction (TSQM-9),^e^ mean (SD)	
Effectiveness	44.9 (14.9)
Convenience	52.3 (18.2)
Global satisfaction	44.5 (13.9)
PD-related QoL (PDQ-8),^d^ mean (SD)	36.9 (19.9)
Freezing of Gait Questionnaires (FOGQ),^g^ mean (SD)	13.9 (5.4)
PD Sleep Scale (PDSS-2)^f^, mean (SD)	21.6 (11.2)
PD Dyskinesia Scale (PDYS-26),^h^ mean (SD)	31.4 (19.6)
Fatigue (PROMIS-F SF),^i^ mean (SD)	58.1 (8.8)
Ability to walk, n (%)	
I am able to walk	27.0 (26.0)
I need help to walk	77.0 (74.0)
PD-related comorbidities, n (%)	
Depression	42.0 (40.4)
Orthostatic/postural hypotension	29.0 (27.9)
PD-related psychosis (hallucination or delusion)	23.0 (22.1)
PD-related dementia (cognitive impairment or decline)	15.0 (14.4)
Key adjunctive PD therapies (yes), n (%)	
Carbidopa/levodopa extended-release tablets	70 (67.3)
Amantadine	33 (31.7)
Rotigotine transdermal patch	32 (30.8)
DBS	22 (21.2)
Apomorphine injections	17 (16.3)
Caregiver support (yes), n (%)	45 (43.3)

**Abbreviations:** DBS, deep brain stimulation; FOGQsa, Freezing of Gait Questionnaire self-administered version; PD, Parkinson's disease; PDSS-2, Parkinson's Disease Sleep Scale; PDQ-8, Parkinson's Disease Questionnaire – Short Form; PDYS-26, Parkinson's disease Dyskinesia Scale; PROMIS-F SF, Patient Reported Outcome Measurement Information System-Fatigue Short Form; QoL, quality of life; SD, standard deviation; TSQM-9, the abbreviated Treatment Satisfaction Questionnaire for Medications; UPDRS, Unified Parkinson's Disease Rating Scale.

a Patients with baseline visit and 36-month visit; 80% PDC logic applied; baseline OFF-time normalized between 3 and 12 h for baseline; last observation carried forward is applied for missing visits between baseline to 36 months; at least 1 follow-up assessment reported after baseline.

b OFF-time was captured in the Modified MDS-UPDRS part IV item 39 and normalized to a 16-h waking day.

c Number of hours of dyskinesia/day was captured in the Modified MDS-UPDRS Part IV item 32 as average hours of dyskinesia in the past 7 days.

d TSQM-9 domains are scored on a scale of 0–100 points, with higher scores indicating greater satisfaction.

e PDQ-8 has 8 items scored on a 0- to 4-point scale, with a higher score indicating worse PD-related QoL.

f PDSS-2 measures PD-related sleep disturbances on a scale of 0–60 with higher scores indicating worse outcomes.

g FOGQsa is a 6-item questionnaire with total scores ranging from 0 to 24, with higher scores indicating more severe freezing of gait.

h PDYS-26 is a 26-item patient-based questionnaire designed to assess the impact of dyskinesia on activities of daily living in PD.

i PROMIS-Short Form v1.0 – Fatigue 8a measures the experience and impact of fatigue, with a range for standardized T-scores of 10–90, with higher scores equating to higher fatigue severity.

### 3.2 PRO outcomes

**Fig. 2:**
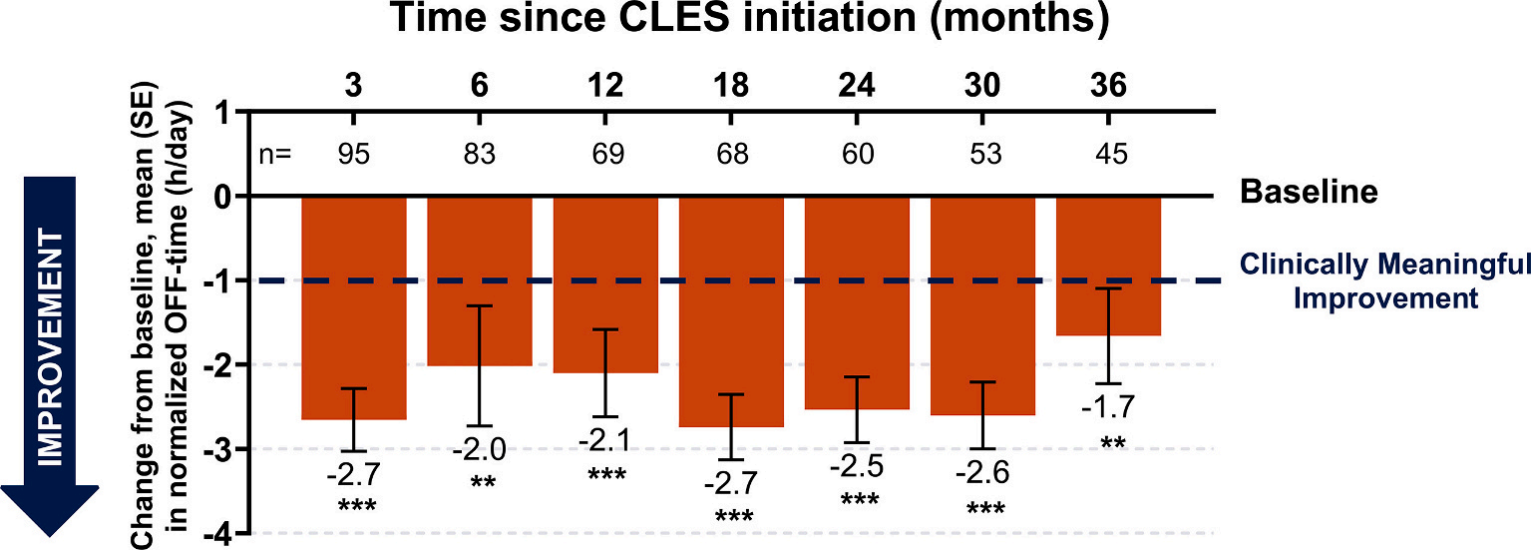
Adjusted estimates of mean change from baseline in normalized patient reported OFF-time across 36 months after CLES initiation. OFF-time was normalized to a 16-h waking day. Mixed models were adjusted for age, sex, ability to walk, number of years since PD diagnosis, and discontinuation status; error bars represent standard error of the mean. The significance level for change from baseline was determined using the one-sample *t-*test. **p < .01; ***p < .001 vs baseline. A reduction of at least 1 h of OFF-time is considered a clinically meaningful improvement. Dashed lines indicate thresholds for meeting defined clinically meaningful improvement from baseline. Abbreviations: CLES, carbidopa/levodopa enteral suspension; h, hour; SE, standard error.

Treatment with CLES resulted in a reduction in the mean (SD) normalized hours of OFF-time per day from 5.7 (3.1) at baseline to 3.0 (3.4) hours as early as the first postbaseline assessment at month 3, to 2.5 [2.8] hours at month 12, to 2.8 (2.9) hours at month 24, and to 3.5 (3.2) hours at month 36. Patients achieved over twice the minimal clinically meaningful improvement in daily OFF-time (ie ≥1 h) within 3 months (mean change: −2.7 h, *p* < .0001) and was sustained through 36 months (−2.2 h, *p* = .004) and were statistically significant from baseline (Fig. 2). Treatment continuation was significantly associated with reduced OFF-time (−1.1 h, *p* = .008).

**Fig. 3:**
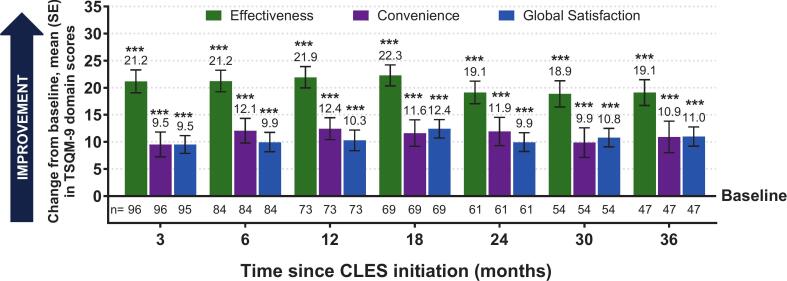
Adjusted estimates of mean change from baseline in treatment satisfaction across 36 months after CLES initiation. Treatment satisfaction was measured across 3 domains (effectiveness, convenience, and global satisfaction) using the TSQM-9. Mixed models were adjusted for age, sex, ability to walk, number of years since PD diagnosis, and discontinuation status. The significance level for change from baseline was determined using the one-sample t-test. *p < .05; **p < .01; ***p < .001 vs baseline. Abbreviations: CLES, carbidopa/levodopa enteral suspension; SE, standard error; TSQM-9, Treatment Satisfaction Questionnaire for Medication-9.

At baseline (before CLES initiation), mean (SD) score for treatment effectiveness using the TSQM-9 was 44.9 (14.9) points, for convenience was 52.3 (18.2), and global satisfaction was 44.5 (13.9). Mean scores for patient satisfaction were improved after CLES initiation at all time points across all three TSQM-9 domains, with the greatest significant improvements for satisfaction with treatment effectiveness through month 36 (ranging from +18.9 to +22.3 points, *p* < .0001, all time points) (Fig. 3**)**. A statistically significant improvement in satisfaction with treatment convenience was reported through month 36 (≥9.0 points, months 3–24, p < .0001; month 36, *p* = .0003). Global satisfaction was also statistically improved from baseline through month 36 (≥9 points; p < .0001). Patients' ability to walk (without an aid) was a significant predictor for improved self-perceived convenience with CLES (+6.0 points; *p* < .05) and global satisfaction (+5.2 points, p < .05). Across all time-points after CLES initiations, patients were generally satisfied with treatment; results for single items for TSQM9 are presented in **Supplemental Fig. 1**.

At baseline, the mean (SD) PDQ-8 score was 36.9 (19.9), and statistically significant improvements were reported after CLES initiation at month 3 (−4.1 [1.7], *p* = .015), month 6 (−4.2 [1.6], *p* = .008), and month 18 (−4.1 [1.5]; *p* = .007) with no worsening of PD-related QoL over time. Patients' ability to walk was a significant predictor for clinically significant improvement in PD-related QoL (reduction in PDQ-8 score of −11.6 [3.6], *p* = .002). At baseline, the mean (SD) FOGQsa score was 13.9 (5.4), with significant improvements observed as early as month 3 (−1.7 [0.5], *p* = .0006) and sustained through month 18 (−2.1 [0.6], *p* = .0007). Patient treatment continuation was a significant predictor for improvement in patients' FOG after CLES initiation (−1.9 [0.8], *p* = .02). Mean (SD) PDSS-2 score at baseline was 21.6 (11.2), and statistically significant improvements from baseline were recorded at month 3 (−2.9 [1.0], *p* = .005) and month 6 (−2.8 [0.9], *p* = .003). Though there were no clinical improvements in sleep quality, the patient-reported average number of hours of sleep increased significantly up to 1.5 (0.4) additional hours/day at 36 months (*p* < .0001). There was a significant decrease in the PROMIS-F scores at month 18 (−1.9 [0.7], *p* = .009). Patients who reported no difficulty walking at baseline showed greater improvement in fatigue (−3.5 [1.7] points, *p* = .004) than those who reported difficulty walking. After adjusting for covariates in the mixed model analysis, the patient-reported average hours of dyskinesia/day using the PDYS-26, significantly decreased at month 3 (−0.7 [0.3], *p* = .03); changes from baseline were not significant at any other timepoints.

### 3.3 Subgroup analysis

In subgroup analyses, 22 patients reported DBS treatment prior to CLES initiation (**Supplemental Table 1**). Similar trends in results were observed in this subgroup population. Clinically meaningful improvements in OFF-time were reported at month 3 (−3.3 h/day) and sustained through 36 months (−3.1 h/day) after CLES initiation (**Supplemental Fig. 2**). Treatment satisfaction improved as early as 3 months and was sustained throughout 36 months. Greatest improvements were reported for treatment effectiveness, followed by global satisfaction with treatment and treatment convenience (**Supplemental Fig. 3**). Slight improvement in PDQ-8 scores was recorded but did not reach statistical significance at any time point. Significant improvements in FOGQsa scores were reported at month 12 (−3.3, *p* = .017) and month 18 (−4.5, *p* = .0016).

## 4 Discussion

PROviDE is the largest long-term, follow-up study evaluating the real-world effectiveness of CLES using PROs in patients with aPD in the US. In this study, CLES provided a significant and clinically meaningful reduction in OFF-time and increased patient-reported treatment satisfaction as early as 3 months after CLES initiation and was sustained throughout 36 months. Clinically meaningful improvements in PD-related QoL were also observed up to 18 months after CLES initiation. Additionally, this long-term follow-up study demonstrated that patients with self-reported DBS who later initiated CLES also experienced clinically meaningful and sustained reduction in OFF-time, improved satisfaction with CLES, and did not experience significant worsening in PD-related QoL.

Results from the PROviDE study are consistent with the findings of other US and multinational studies of the long-term treatment benefit of CLES on OFF-time in aPD. In the GLORIA registry study, patients initiating CLES experienced significant and sustained reductions in OFF-time for the duration of the 24-month study (−4.1 h/day) [22]. A post hoc analysis from the GLORIA registry showed statistically significant improvements in ADL, motor symptoms, and nonmotor symptoms in patients receiving CLES monotherapy compared with polytherapy [23]. Similarly, and consistent with the magnitude of improvement observed in PROviDE, significant improvements in OFF-time were also maintained over 3 years (−3.3 h/day; *p* < .001) in the DUOGLOBE, a multinational, observational analysis of the long-term effectiveness of CLES in patients aPD [10]. PROviDE provides additional real-world data to support the growing body of evidence on the impact of CLES on motor outcomes in patients with aPD. Notably, results are also comparable to those observed with continuous subcutaneous infusion of LDp/CDp, which has similarly demonstrated OFF-time improvements [24].

PROviDE is among the few studies to directly assess patient-reported treatment satisfaction with CLES therapy in aPD. This study demonstrated that patients were more satisfied with CLES relative to their previous PD treatments, which had primarily involved oral pills. Importantly, these results show that patients successfully adapted to using a pump for their PD management, highlighting their acceptance of such approach. Although caregiver data were not collected in this real-world study, the potential influence of caregiver involvement on adherence, tolerability, and PROs should be considered in future studies. Patient satisfaction with both treatment effectiveness and convenience did not decline over 36 months. The perceived high levels of effectiveness suggest that patients believe the treatment works well for them, even though some other PROs showed less improvement over time. Studies shows that greater treatment satisfaction is associated with higher treatment compliance and persistence [25]. In addition to utilizing patient satisfaction to inform treatment decisions and product lifecycle management, understanding long-term changes in treatment satisfaction and the potential importance of caregiver support may help predict the likelihood of continued treatment adherence and improved QoL.

In the PROviDE study, statistically significant improvements in PDQ-8 were observed at 3, 6, and 18 months following CLES initiation with no worsening of QoL over time. Other long-term studies have also shown that previous improvements in measures of QoL and ADL with CLES are not sustained over time [26], [27], suggesting that these outcome measures may be less sensitive to detect changes throughout treatment duration. It is plausible that patients experience the greatest improvements soon after initiating treatment and are less aware of additional progression once their symptoms are well managed. This phenomenon is known as a response shift; individuals with PD often “normalize” their experiences or adapt to their symptoms and adjusted standards of QoL. As a result, over longer periods, changes in perceived QoL may appear smaller or may return to baseline levels. From a clinical perspective, sustained treatment benefit is an important aspect of long-term PD management.

FOG in aPD is a challenging clinical symptom to assess due to its episodic nature; it is often associated with an increased risk of falls and loss of mobility [28]. Significant improvements in FOG occurred early in the PROviDE study, however, these benefits were not sustained over time, which may be attributable to the limitations of the 16-h CLES treatment. Because FOG is a multifactorial symptom and may not be fully explained by dopaminergic mechanisms alone, the early improvement observed with CLES may reflect reduced OFF-related gait dysfunction rather than a direct effect on FOG itself; however, this will need to be further evaluated in future studies [28]. It is possible that although some patients may experience early improvement in FOG, benefits may not be sustained long-term and individual response varies [29].

Sleep disturbances are highly prevalent among people with aPD and can have a significant negative impact on their QoL. The standard 16-h CLES infusion has been previously shown to provide improvements in sleep [30], and studies examining CLES administered over 24 h, have reported further improvements in sleep symptoms [31]. Significant improvements in sleep quality also occurred early in the PROviDE study but were not sustained over time, possibly reflecting the limitations of a 16-h waking-day continuous infusion. With the availability of new continuous subcutaneous 24-h continuous infusions, such as LDp/CDp, there is potential for greater and more durable improvements in sleep for patients with aPD.

There is limited evidence available for patients on CLES who were previously treated with DBS. Most of the studies examining these patients are case series [14], [15], supporting the use of CLES in patients receiving DBS who continue to experience refractory symptoms after optimization or long-term therapy. No major difficulties regarding prior DBS lead implantation were reported throughout the duration of this study. As the requirement for oral medication increases in DBS-treated patients, CLES may offer an advantage over oral levodopa because the fluctuating plasma levels of orally administered levodopa may lead to severe dyskinesia and there is less pill burden. Concomitant DBS and CLES may occur, with the relative contribution of DBS and CLES to the patient's symptom control adjusted as needed, for an individualized therapy. Our results are comparable to other studies on CLES in patients with a history of DBS or in combination with DBS showing a reduction in OFF-time, increased treatment satisfaction, and improved QoL. Additional research is needed to further understand patient characteristics and treatment modalities of using CLES in patients previously or concurrently treated with DBS.

### 4.1 Limitations

This study provides important real-world data on the use of CLES to treat patients with aPD, however there are certain limitations inherent in observational prospective studies that should be considered when interpreting these results, including the open-label design and lack of a control group. There may be subjectivity in patient self-completion of the PROs in the absence of clinician assessment of the same symptoms, which may introduce self-reporting recall bias. PD treatment was managed by independent physicians, safety data were not collected, and all evaluations were done on the phone/online, reducing samples over time and continuations of study/treatment. The results should be interpreted with caution due to potential selection bias including the substantial attrition observed during follow-up and missing data points that may not be fully addressed with the LOCF method. The observed outcomes may therefore be enriched for a subgroup of patients who tolerated and continued the treatment, and consequently, the results may be overly influenced by good responders, limiting the generalizability of the findings*.* However, compared to claims-based studies where a low number of patients are analyzed over a year, the sample for this study can be considered relatively large. In addition, a substantial number of patients discontinued the study prematurely or were not included in the mixed model analysis due to stringent inclusion criteria, potentially limiting the interpretation of the current study's results. For the subgroup analysis of patients with prior reported DBS treatment, it is unknown whether CLES was used concurrently with ongoing DBS or as a subsequent therapy after DBS discontinuation; therefore, the independent contribution of each therapy to the observed outcomes cannot be distinguished and the observed effects of CLES alone can only be extrapolated.

## 5 Conclusions

In this real-world study, CLES initiation led to early and sustained clinically meaningful reductions in OFF-time over 36 months among patients with aPD living in the US. Despite switching their PD treatment regimen from other treatments to CLES, patients reported higher levels of satisfaction with CLES and continued to report increased satisfaction with prolonged use of CLES. The high levels of satisfaction with prolonged CLES further support the long-term utilization of CLES in patients with aPD with motor symptoms fluctuations. Significant improvements in PD-related QoL were observed up to 18 months after initiation of CLES. By providing sustained long-term effects in reducing hours of OFF-time, improving treatment satisfaction, and maintaining PD-related QoL in patients previously treated with DBS, CLES may be a viable treatment option for patients who are suboptimally managed on DBS.

## Data sharing

AbbVie is committed to responsible data sharing regarding the clinical trials we sponsor. This includes access to anonymized, individual, and trial-level data (analysis data sets), as well as other information (eg, protocols, clinical study reports, synopses, or statistical analysis plans), as long as the trials are not part of an ongoing or planned regulatory submission. These clinical trial data can be requested by any qualified researchers who engage in rigorous, independent, scientific research, and will be provided following review and approval of a research proposal, Statistical Analysis Plan (SAP), and execution of a Data Use Agreement (DUA). Data requests can be submitted at any time after approval in the US and Europe and after acceptance of this manuscript for publication. The data will be accessible for 12 months, with possible extensions considered. For more information on the process or to submit a request, visit the following link: https://vivli.org/ourmember/abbvie/ then select “Home”.

## CRediT authorship contribution statement

**Rajesh Pahwa:** Writing – review & editing, Methodology. **Stuart H. Isaacson:** Writing – review & editing, Methodology. **David Cella:** Writing – review & editing, Methodology. **Sandeep Thakkar:** Writing – review & editing, Methodology. **Prasanna L. Kandukuri:** Writing – review & editing, Visualization, Validation, Software, Methodology, Formal analysis, Data curation. **Connie H. Yan:** Writing – review & editing, Writing – original draft, Validation, Supervision, Project administration, Methodology, Formal analysis, Data curation, Conceptualization. **Pavnit Kukreja:** Writing – review & editing, Methodology, Conceptualization. **Irene Pan:** Writing – review & editing, Methodology. **Jae Lee:** Writing – review & editing, Validation, Supervision, Project administration, Methodology, Formal analysis, Data curation, Conceptualization. **Jason Aldred:** Writing – review & editing, Methodology.

## Funding statement

This study was funded by AbbVie Inc. AbbVie participated in the study design, research, data collection, analysis and interpretation of data, writing, reviewing, and approving the publication. All authors listed have made a substantial, direct, and intellectual contribution to the work. All authors had access to data results, and participated in the development, review, and approval of this abstract. No honoraria or payments were made for authorship.

## Declaration of competing interest

The authors declare the following financial interests/personal relationships which may be considered as potential competing interests: **Rajesh Pahwa** reports grants from Abbott, AbbVie, Alexza, Annovis, Biogen, Bluerock, Bukwang, Cerevel, Global Kinetics, Jazz, the Michael J Fox Foundation, NeuroDerm, Neuraly, the Parkinson's Foundation, Praxis, Roche, Sage, Scion, Sun Pharma, Supernus Pharmaceuticals, UCB, and Voyager; and consulting fees from Abbott, AbbVie, ACADIA, Acorda, Allevion, Amneal, Artemida, BioVie, CalaHealth, Convatec, Global Kinetics, Inbeeo, Insightec, Jazz, Kyowa, Lundbeck, Merz, Neurocrine, NeuroDerm, Ono, PhotoPharmics, Sage, Sunovion, Supernus, UCB, and Wren. **Stuart Isaacson** has received Honoraria for CME, consultant, research grants, and/or promotional speaker on behalf of: AbbVie, Acadia, Acorda, Alexza, Amneal, Amylyx, Bial, Biogen, BioVie, Britannia, Cala, Cerevel, CND Life Sciences, Eli Lilly, Enterin, Esteve, Fasikl, Global Kinetics, Jazz, Kyowa Kirin, Lundbeck, Merz, Michael J. Fox Foundation, Mitsubishi Tanabe, Neurocrine, Neuroderm, Novartis, Ono, Parkinson Study Group, Pharma2B, Praxis, Revance, Roche, Rune, Sage, Sanofi, Scion, Stoparkinson, Supernus, Teva, Theravance, UC. **David Cella** has been a consultant and received honoraria from AbbVie, AstraZeneca, Bristol-Myers Squibb (BMS), GlaxoSmithKline (GSK), Novartis, Pfizer, Ipsen, Asahi Kasei, Pled Pharma, Analysis Group, and Merck/EMD Serono, and he has received research funding from NIH, AbbVie, GSK, Novartis, BMS, Pfizer, Pled Pharma, Ipsen, and Biogen via the National Quality Forum. **Sandeep Thakkar** has the following to disclose concerning possible financial or personal relationships with commercial entities that may have a direct or indirect interest in the subject matter of this presentation: has received consulting fees from AbbVie and a speaker on behalf of Medtronic, AbbVie, Teva, UCB, Neurocrine. **Prasanna** L. **Kandukuri**, **Connie H. Yan**, **Pavnit Kukreja,** and **Jae Lee** are employees of AbbVie and may own stock options in the company. **Irene Pan** is an employee of UBC and may own stocks/shares in the company. **Jason Aldred** is a study investigator and has received honoraria from AbbVie, Allergan, Teva, US World Meds, Medtronic, and Abbot. He is an investigator in studies sponsored by AbbVie, AC Immune, Annovis, Aptinyx, AstraZeneca, Atara, Athira, Biogen, BioVie, 10.13039/100008497Boston Scientific, Celgene, Cerevance, Cerevel, Denali, EIP, Eli Lilly, Impax, Inhibikase, IRL Therapeutics, Merz, Neuraly, Neurocrine, Neuroderm, Novartis, PD Gene/PSG, Praxis, Revance, Roche/Genentech, Sage, Sanofi/Genzyme, Scion Neurostem, Takeda, Theravance, Triplet/HSG, and UCB. He is also a scientific advisor for AbbVie, has been a consultant and received honorarium from AbbVie, Acorda, Adamas, Allergan, Boston Scientific, Teva, Medtronic, and US World Meds. He has also received research support from NINDS, Abbott, AbbVie, Acadia, Biogen, Boston Scientific, Denali, Impax/Amneal, Sunovion, Neuroderm, Novartis, and Theravance.
