# Supplementary material for: Long-term real-world effectiveness of carbidopa/levodopa enteral suspension after 36 months of treatment initiation in advanced Parkinson's disease in the US: final results from the PROviDE study
Source: Clin Park Relat Disord. 2026 Jul 26;15:100488. doi: 10.1016/j.prdoa.2026.100488 (PMC13452392; doi:10.1016/j.prdoa.2026.100488)
Supplement: Supplementary file 1 [file mmc1.docx]

**TITLE: Long-Term Real-World Effectiveness of Carbidopa/Levodopa Enteral Suspension After 36 Months of Treatment Initiation in Advanced Parkinson’s Disease in the US: Final Results From the PROviDE Study**

**SUPPLEMENTAL MATERIAL (1 Table and 3 Figures)**

| **Supplemental Table 1.** Baseline demographic and clinical characteristics of prior DBS patients ^a^ | |
| --- | --- |
| **Characteristic** | **Patients  N = 22** |
| Age, years, mean (SD) | 67.6 (8.7) |
| Male, n (%) | 12.0 (54.6) |
| Years since PD diagnosis, mean (SD) | 14.7 (7.1) |
| Hours of OFF-time^b^, mean (SD) | 6.0 (3.3) |
| Hours of dyskinesia per day,^c^ mean (SD) | 1.8 (1.8) |
| Treatment satisfaction (TSQM-9),^d^ mean (SD) |  |
| Effectiveness | 45.7 (13.8) |
| Convenience | 52.8 (18.9) |
| Global satisfaction | 45.2 (13.7) |
| PD-related QoL (PDQ-8),^e^ mean (SD) | 41.0 (18.5) |
| PD Sleep Scale (PDSS-2),^f^ mean (SD) | 15.6 (9.4) |
| Freezing of Gait Questionnaires (FOGQ),^g^ mean (SD) | 13.9 (5.3) |
| PD Dyskinesia Scale (PDYS-26),^h^ mean (SD) | 31.6 (20.7) |
| Fatigue (PROMIS-F SF),^i^ mean (SD) | 57.6 (11.2) |
| Ability to walk, n (%) |  |
| I am able to walk | 4.0 (18.2 ) |
| I need help to walk (i.e. cane or walker) | 14.0 (63.6 ) |
| I have limited ability to walk and primarily use wheelchair | 4.0 (18.2 ) |
| Key adjunctive PD therapies (yes), n (%) |  |
| DBS | 22.0 (100) |
| Carbidopa/levodopa extended-release tablets | 19.0 (86.4) |
| Amantadine | 7.0 (31.8) |
| Apomorphine injections | 6.0 (27.3) |
| Rotigotine transdermal patch | 4.0 (18.2) |
| PD-related comorbidities, n (%) |  |
| Depression | 9.0 (40.9) |
| Orthostatic/postural hypotension | 5.0 (22.7) |
| PD-related psychosis (hallucination or delusion) | 1.0 (4.5) |
| PD-related dementia (cognitive impairment or decline) | 2.0 (9.1) |
| **Abbreviations:** DBS, deep brain stimulation; FOGQsa, Freezing of Gait Questionnaire self-administered version; PD, Parkinson’s disease; PDSS-2, Parkinson’s Disease Sleep Scale; PDQ-8, Parkinson’s Disease Questionnaire – Short Form; PDYS-26, Parkinson Disease Dyskinesia Scale; PROMIS-F SF, Patient Reported Outcome Measurement Information System-Fatigue Short Form; QoL, quality of life; SD, standard deviation; TSQM-9, the abbreviated Treatment Satisfaction Questionnaire for Medications; UPDRS, Unified Parkinson’s Disease Rating Scale. | |
| ^a^ Patients with previous use of DBS prior to initiation of CLES; baseline visit and 36-month visit, 80% PDC logic applied; baseline OFF-time normalized between 3-12 hours for baseline; last observation is carried forward applied for missing visits between baseline to 36 months. | |
| ^b^ OFF-time was captured in the Modified UPDRS part IV item 39 and normalized to a 16-hour waking day. | |
| ^c^ Number of hours of dyskinesia/day was captured in the Modified UPDRS Part IV item 32 as average hours of dyskinesia in the past 7 days.  ^d^ PDQ-8 has 8 domains scored on a 0- to 4-point scale and presented as a percentage, with a higher score indicating worse PD-related QoL. | |
| ^e^ TSQM-9 is on a scale of 0–100 points, with higher scores indicating greater satisfaction, and is measured across three domains. | |
| ^f^ PDSS-2 measures PD-related sleep disturbances on a scale of 0-60 with higher scores indicating worse outcomes. | |
| ^g^ FOGQsa is a 6-item questionnaire with total scores ranging from 0 to 24, with higher scores indicating more severe freezing of gait. | |
| ^h^ PDYS-26 is a 26-item patient-based questionnaire designed to assess the impact of dyskinesia on activities of daily living in PD. | |
| ^i^ PROMIS-Short Form v1.0 – Fatigue 8a measures the experience and impact of fatigue, with a range for standardized T-scores of 10-90, with higher scores equating to higher fatigue severity. | |

**Supplemental Figure 1:** Patient-reported treatment satisfaction with CLES across 36 months. Treatment satisfaction was measured for 9 items across 3 domains (effectiveness, convenience, and global satisfaction) using the TSQM-9 (TSQM01-09). Instruction for patients: please take some time to think about your level of satisfaction or dissatisfaction with the medication you are taking for PD over the last two to three weeks, or since you last used it. TSQM-9 single items were assessed using either a 7- or 5-point Likert rating scale (eg, 7-point scale for TSQM01-03 and TSQM-09: “satisfied” = “extremely satisfied + very satisfied + satisfied + somewhat satisfied”, and “dissatisfied” = “dissatisfied + very dissatisfied + extremely dissatisfied”, for TSQM04-05: ”easy” = “extremely easy + very easy + easy + somewhat easy”, and “difficult” = “difficult + very difficult + extremely difficult”, for TSQM06: ”convenient” = “extremely convenient + very convenient + convenient + somewhat convenient”, and “inconvenient” = “inconvenient + very inconvenient + extremely inconvenient”; and 5-point scale for TSQM-07: ”confident” = “extremely confident + very confident + somewhat confident”, “not confident” = “a little confident + not at all confident”, and for TSQM-08: ”certain” = “extremely certain + very certain + somewhat certain”, and “not certain” = “a little certain + not at all certain”). Abbreviations: CLES, carbidopa/levodopa enteral suspension; PD, Parkinson’s disease; TSQM-9, Treatment Satisfaction Questionnaire for Medication-9.

**Supplemental Figure 2:** Adjusted estimates of mean change from baseline in normalized patient reported OFF-time for patients with prior DBS after CLES initiation across 36 months. OFF-time was normalized to a 16-hour waking day. Mixed models were adjusted for age, sex, ability to walk, number of years since PD diagnosis, and discontinuation status; error bars represent standard error of the mean. The significance level for change from baseline was determined using the one-sample t test. *p<.05; **p<.01; ***p<.001 vs baseline. A reduction of at least 1 hour of OFF-time is considered a clinically meaningful improvement. Dashed lines indicate thresholds for meeting defined clinically meaningful improvement from baseline. Abbreviations: CLES, carbidopa/levodopa enteral suspension; h, hour; SE, standard error.

**Supplemental Figure 3:** Adjusted estimates of mean change from baseline in treatment satisfaction for patients with prior DBS after CLES initiation across 36 months. Treatment satisfaction was measured across 3 domains (effectiveness, convenience, and global satisfaction) using the TSQM-9. Mixed models were adjusted for age, sex, ability to walk, number of years since PD diagnosis, and discontinuation status. The significance level for change from baseline was determined using the one-sample t test. *p<.05; **p<.01; ***p<.001 vs baseline. Abbreviations: CLES, carbidopa/levodopa enteral suspension; SE, standard error; TSQM-9, Treatment Satisfaction Questionnaire for Medication-9.
